# Optimization of In-Situ Growth of Superconducting Al/InAs Hybrid Systems on GaAs for the Development of Quantum Electronic Circuits

**DOI:** 10.3390/ma18020385

**Published:** 2025-01-16

**Authors:** Magdhi Kirti, Máté Sütő, Endre Tóvári, Péter Makk, Tamás Prok, Szabolcs Csonka, Pritam Banerjee, Piu Rajak, Regina Ciancio, Jasper R. Plaisier, Pietro Parisse, Giorgio Biasiol

**Affiliations:** 1 CNR-IOM—Istituto Officina dei Materiali, Consiglio Nazionale delle Ricerche, 34149 Trieste, Italy; magdhi.kirti27@gmail.com (M.K.); pritban@dtu.dk (P.B.); piu.rajak@gmail.com (P.R.); regina.ciancio@areasciencepark.it (R.C.); parisse@iom.cnr.it (P.P.); 2 Department of Physics, University of Trieste, 34128 Trieste, Italy; 3 Department of Physics, Institute of Physics, Budapest University of Technology and Economics, H-1111 Budapest, Hungary; suto.mate@ttk.bme.hu (M.S.); tovari.endre@ttk.bme.hu (E.T.); makk.peter@ttk.bme.hu (P.M.); tamas.prok@edu.bme.hu (T.P.); csonka.szabolcs@ttk.bme.hu (S.C.); 4 MTA-BME Superconducting Nanoelectronics Momentum Research Group, H-1111 Budapest, Hungary; 5 HUN-REN Centre for Energy Research, Institute of Technical Physics and Materials Science, Konkoly Thege Miklós út 29-33, 1121 Budapest, Hungary; 6 MTA-BME Correlated van der Waals Structures Momentum Research Group, H-1111 Budapest, Hungary; 7 Department of Physics, Balurghat College, Dakshin Dinajpur, West Bengal 733101, India; 8 Area Science Park, 34149 Trieste, Italy; 9 Elettra-Sincrotrone Trieste S.C.p.A., 34149 Trieste, Italy; jasper.plaisier@elettra.eu

**Keywords:** quantum computation, InAs 2DEG, aluminum, metamorphic growth

## Abstract

Hybrid systems consisting of highly transparent channels of low-dimensional semiconductors between superconducting elements allow the formation of quantum electronic circuits. Therefore, they are among the novel material platforms that could pave the way for scalable quantum computation. To this aim, InAs two-dimensional electron gases are among the ideal semiconductor systems due to their vanishing Schottky barrier; however, their exploitation is limited by the unavailability of commercial lattice-matched substrates. We show that in situ growth of superconducting aluminum on two-dimensional electron gases forming in metamorphic near-surface InAs quantum wells can be performed by molecular beam epitaxy on GaAs substrates with state-of-the-art quality. Adaptation of the metamorphic growth protocol has allowed us to reach low-temperature electron mobilities up to 1.3 × 10^5^ cm^2^/Vs in Si-doped InAs/In_0.81_Ga_0.19_As two-dimensional electron gases placed 10 nm from the surface with charge density up to 1 × 10^12^/cm^2^. Shubnikov-de Haas oscillations on Hall bar structures show well-developed quantum Hall plateaus, including the Zeeman split features. X-ray diffraction and cross-sectional transmission electron microscopy experiments demonstrate the coexistence of (011) and (111) crystal domains in the Al layers. The resistivity of 10-nm-thick Al films as a function of temperature was comparable to the best Al layers on GaAs, and a superconducting proximity effect was observed in a Josephson junction.

## 1. Introduction

The path to the implementation of reliable and fault-tolerant quantum computers relies on the availability of suitable material platforms. In this regard, the coupling of a semiconducting material with a superconductor allows the realization of a hybrid system in which certain properties of the superconductor can be harnessed in conjunction with the tunability offered by semiconducting materials [1,2,3,4,5]. The transport properties and the formation of localized states at the boundaries of these hybrid systems have gained much attention in recent years due to their potential applications in the field of quantum computing [6,7,8,9].

In these hybrid systems, growth of high-quality superconducting thin films with transparent interfaces to a low-D semiconductor is expected to improve the coherence time of supra-semi qubits and enable strong qubit-qubit coupling [10]. Therefore, these semiconductor-superconductor hybrid systems used as a platform for qubits, such as Andreev qubits, gatemons, or topological systems such as Kitaev chains, Kitaev-transmons, etc., are among the promising candidates for quantum computation applications [11,12,13,14,15]. The basic components are (a) narrow strips of semiconductor two-dimensional electron gases (2DEGs) with a strong spin−orbit coupling, (b) a transparent interface between the superconductor and semiconductor, and (c) proximity-induced superconductivity in the semiconductor with an external magnetic field to reach the topological superconducting regime [10]. Therefore, the ideal candidates for the constituent materials supporting these qubits with superconducting elements are low-bandgap semiconductors such as InAs, possessing a large Lande’s g-factor and strong spin-orbit coupling. Moreover, aluminum as a superconducting film has a long superconducting coherence length and is compatible with standard fabrication techniques. The controlled proximity effect with superconductors is the critical aspect for many applications [16,17], and it can best be implemented by separating the 2DEGs in InAs quantum wells from epitaxially grown aluminum by a thin enough InGaAs barrier [18,19,20].

In recent years, hybrid systems consisting of shallow InAs 2DEGs coupled to Al on different substrates have been investigated. The highest reported 2DEG mobility was reached on almost lattice-matched GaSb substrates, allowing growth of InAs quantum wells (QWs) with thicknesses up to 24 nm and low-T electron mobility µ ~2 × 10^6^ cm^2^/Vs [21]. The growth on the more technologically relevant and resistive InP and GaAs wafers limits the InAs QW thickness to a few nm due to strain, with mobility (µ) up to ~10^6^ cm^2^/Vs and ~7.1 × 10^5^ cm^2^/Vs on InP and GaAs, respectively [22,23]. Among these latter substrates, GaAs substrates would represent the best choice, despite the higher lattice mismatch to InAs (7%) with respect to InP (3%), as they are more resistive (which is an advantage for high-frequency operations) and less costly.

The above mobilities were reached in deep quantum wells with the 2DEG located about 100 nm below the top surface, making them inadequate for proximity-induced superconductivity studies. As the 2DEG approaches the surface, surface scattering decreases the electron mobility [24]. Therefore, it is important to investigate protocols to reduce mobility degradation in near-surface 2DEGs while preserving the ease with which superconductivity can be induced.

In the initial part of our study, we have optimized shallow InAs 2DEG structures grown on GaAs substrates using molecular beam epitaxy (MBE) and performed low-T transport studies on these samples. 1D Poisson-Schrödinger (PS) simulations were used to optimize the thickness of the top In_0.81_Ga_0.19_As barrier in a way to guarantee that the electron wavefunction approaches the surface while still preserving a high enough mobility to ensure ballistic transport. In the second part of the paper, we have optimized the in situ Al layer deposition inside the MBE reactor in order to obtain continuous Al films on the InAs 2DEGs [25]. Al deposition is conformal to the underlying semiconductors, preserving the cross-hatched pattern typical for metamorphic growth. X-ray diffraction (XRD) and cross-sectional transmission electron microscopy (TEM) experiments showed evidence for a crystalline structure, mostly oriented in the [011] direction. Resistivity measurements as a function of temperature gave values comparable to the best single-crystal Al film grown on GaAs. Josephson junctions were fabricated on our InGaAs/InAs 2DEG with epitaxial Al, which exhibited superconducting proximity effects [26].

## 2. Material and Methods

**Figure 1:**
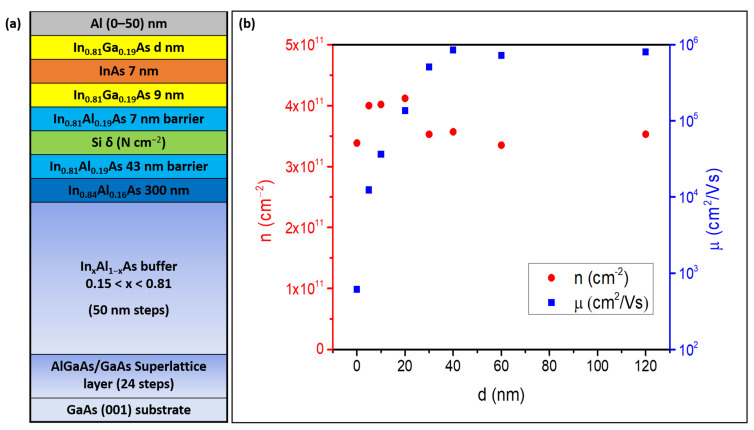
(**a**) Schematics of the growth sequence of the Al-InAs/GaAs hybrid system. (**b**): low-temperature (T = 4.2 K) electron charge density and mobility in undoped InAs 2DEG as a function of *d* (distance of QW from surface).

Solid source MBE was used to grow In_0.81_Al_0.19_As/In_0.81_Ga_0.19_As/InAs QWs on a semi-insulating GaAs (001) substrate. The metamorphic QW region was grown over a strain-relieving step-graded In_x_Al_1-x_As buffer terminated with a 300 nm thick In_0.84_Al_0.16_As layer to ensure optimal strain relaxation and maximize the low-T electron mobility in deep 2DEGs [23]. The QW region consists of a 50 nm thick In_0.81_Al_0.19_As barrier, followed by a 9 nm In_0.81_Ga_0.19_As, 7 nm InAs, and a top In_0.81_Ga_0.19_As barrier whose thickness *d* varied from 120 to 0 nm as shown in Figure 1a. After setting appropriate values for *d* as discussed below, the charge density in shallow QWs was tuned by Si δ-doping at 7 nm in the lower In_0.81_Al_0.19_As barrier.

**Figure 2:**
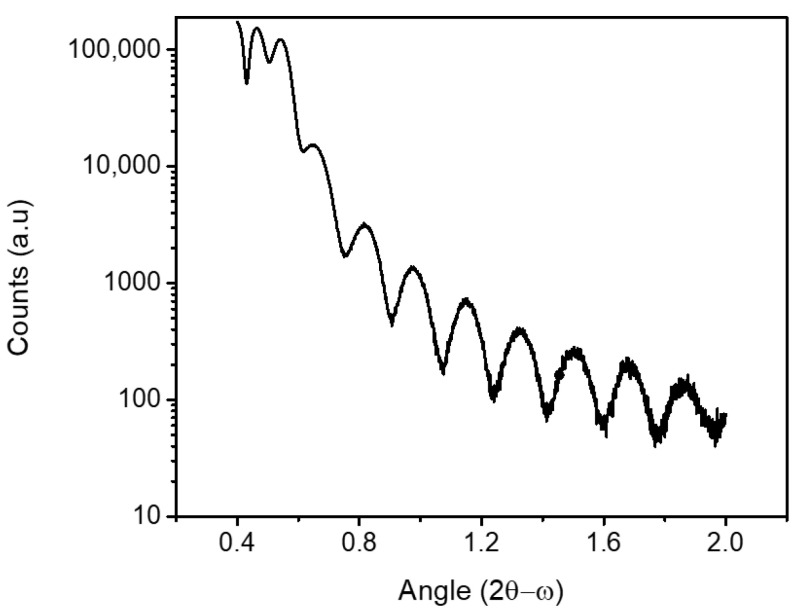
X-ray reflectivity (XRR) of 50 nm thick Al on the InAs/GaAs heterostructures.

After optimization of *d* and Si doping, Al thin films were deposited in situ on shallow InAs QW structures inside the MBE system without exposing the sample surface to air. This ensures that the Al is deposited on an oxide-free, as-grown semiconductor surface while guaranteeing at the same time the best metal material quality. Aluminum was deposited after letting the system pump down the residual arsenic overnight. To prevent aluminum from de-wetting after semiconductor growth, samples were cooled down to about −50 °C before deposition by switching off the manipulator heater and idling the effusion cells [27,28,29]. The C-type manipulator thermocouple was calibrated for temperatures in the range from −170 to +125 °C following Ref. [30]. Although the absolute temperature value on the wafer surface may deviate from the thermocouple reading, this method nevertheless provides a calibration for sample-to-sample reproducibility. Different samples were grown with aluminum layer thicknesses ranging from 10 to 50 nm. Al thickness was calibrated within 2% either by a step profilometer after etching the film or with X-ray reflectivity (XRR) on a nominally 50-nm-thick film deposited directly on GaAs. Figure 2 shows the XRR curve where the Kiessig fringe spacing gives an Al thickness of 50 ± 1 nm. The persistence of oscillations is a signature of the flatness of the top surface and of the bottom Al interface [31].

The properties of 50-nm-thick Al/In_0.81_Ga_0.19_As/InAs samples were compared with those of equivalent Al films grown directly on GaAs (001) epitaxial surfaces with the same procedure.

To benchmark the quality of in situ aluminum growth, we tried deposition on the InAs/GaAs heterostructures using three different ex-situ procedures:

- S-1: After the growth of InAs/GaAs, As_4_ capping was performed in situ in the MBE to protect the semiconductor surface from oxidation, followed by air exposure and then re-insertion into the MBE chamber. The As_4_ is then decapped at around 350 °C, followed by 50 nm Al growth in the MBE with the protocol described above with pressure around 10^−12^ mbar.
- S-2: The InAs/GaAs sample was As-capped and exposed to air as in S-1 and then inserted in a sputtering chamber followed by As_4_ decapping and 50 nm Al growth at room temperature with a background pressure around 10^−7^ mbar.
- S-3: The InAs/GaAs (without the capping layer) was oxidized by air exposure and then inserted in the sputtering chamber where 50 nm Al was grown as in S-2.

Atomic Force Microscopy topographic images were acquired in contact mode (AC mode) with an MFP-3D instrument (Asylum Research/Oxford Instruments) using a silicon cantilever (NSC36 Mikromasch, radius of curvature <10 nm, spring constant 0.6–2 N/m). Images were analyzed with Gwyddion software Version 2.56 [32] to extract the RMS roughness of the different samples. The structural properties of the films were analyzed with XRD at the MCX beamline of Elettra Synchrotron (Trieste, Italy) using a Huber high-resolution X-ray diffractometer at an X-ray energy of 8 keV [33]. High-resolution transmission electron microscopy (HR-TEM) experiments were performed in a JEOL 2010 UHR field emission gun microscope operated at 200 kV, with a measured spherical aberration coefficient Cs of 0.47 ± 0.01 mm. Cross-sectional TEM specimens were prepared using a combination of typical mechanical polishing, dimpling, and ion milling techniques.

Hall measurements on bare 2DEGs were performed at 4.2 K on ~4 × 4 mm^2^ van der Pauw (vdP) structures using a four-terminal conventional lock-in technique with an excitation current of 2 µA at 20 Hz. Selected samples were fabricated in a Hall Bar (HB) geometry with the length/width aspect ratio of 1:2. Four-terminal resistivity measurements of Al layers were completed on ~1 × 4 mm^2^ stripes from 4.2 K to 300 K. Josephson junctions (JJs) with lengths of a few hundred nm were fabricated on InAs/Al platforms using electron beam lithography (EBL), wet etching, electron beam evaporation, and thermal Atomic Layer Deposition (ALD) for a 50-nm-thick gate dielectric Al_2_O_3_. Measurements of the JJs were performed in a dilution flow refrigerator at 35 mK base temperature using standard lock-in technique and DC current-voltage I(V) measurements with an NI USB 6341 Multifunction DAQ. To fine-tune the magnetic field, we used a Yokogawa GS200 DC voltage/current source.

## 3. Results and Discussions

### 3.1. Optimization of InAs QW Structures

As discussed earlier, to obtain high-quality semiconductor/superconductor nano-systems we need to achieve proximity-induced superconductivity. Two important factors for a good proximity effect are a small enough distance of the InAs 2DEGs from the surface and a high enough electron mobility. To find the best compromise between these conflicting requirements, we have investigated the effect of the thickness *d* of the In_0.81_Ga_0.19_As barrier separating the 7-nm InAs QW from the surface in undoped 2DEGs. Figure 1b shows the charge density *n* and electron mobility µ at 4K as a function of *d* in the 0–120 nm range on vdP structures. It can be seen that µ is independent of *d* down to about 40 nm at values around µ = 8 × 10^5^ cm^2^/Vs, consistent with what we previously found on similar samples after optimization of the buffer layer [23]. Mobility then decreases sharply to 3.6 × 10^4^ cm^2^/Vs at *d* = 10 nm, 1.2 × 10^4^ cm^2^/Vs at *d* = 5 nm^,^ and 6.1 × 10^2^ cm^2^/Vs at *d* = 0 nm (i.e., without the top InGaAs barrier, with the InAs QW reaching the surface and forming an about 1.5 nm-thick native oxide [34]). Conversely, *n* is independent of *d* in the whole range, at values of 3–4 × 10^11^ cm^−2^. Mobility degradation as the top barrier thickness is made thinner is attributed to the strengthening of remote Coulomb scattering on charges trapped at the semiconductor/oxide interface forming at the top surface [35].

**Figure 3:**
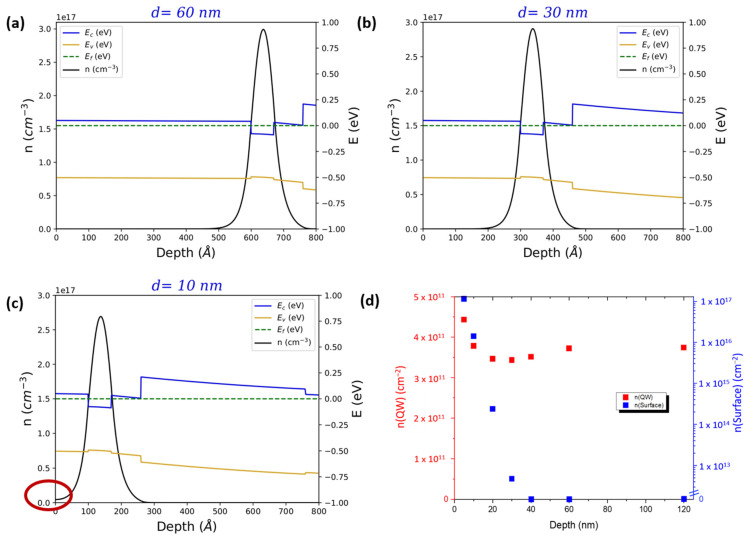
(**a**–**c**) 1D Poisson-Schrödinger simulations at various thicknesses *d* of the top In_0.81_Ga_0.19_As barrier (60 nm, 30 nm, and 10 nm, respectively). Valence and conduction band profiles are indicated in orange and blue, while 3D electron density is indicated in black. The red oval in panel (**c**) indicates the onset of electron population at the surface. (**d**) Electron charge density in the quantum well and 3D density at the surface estimated with PS simulations. The background doping in the In_0.81_Al_0.19_As barriers was set to 4 × 10^16^ cm^−3^, while the value of the Fermi level E_F_ was pinned 15 meV below the conduction band edge at the surface.

These findings can be interpreted in the light of one-dimensional PS simulations performed as a function of *d* in the same range [36]. In the simulations we set an n-type background doping of *n_B_* = 4 × 10^16^ cm^−3^ in the In_0.81_Ga_0.19_As barriers, while the Fermi level at the surface E_F_ was pinned 15 meV below the conduction band minimum. Conduction and valence band edges, as well as the 3D electron density profiles, are shown in Figure 3a–c for three representative values of *d*. The chosen values of *n_B_* and E_F_ ensured that *n* in the InAs QW remains constant at around 3.5–4 × 10^11^ cm^−2^ independently of *d*, as can be seen in Figure 3d (red squares). Note that *n_B_* is slightly larger and E_F_ slightly smaller than what we found in equivalent systems with an In concentration x = 0.75, where the electron density in the QW decreased at depths lower than 150 nm due to Fermi level pinning at the surface [37]. This is consistent with the fact that InGaAs alloys tend to become more n-type as the In content increases [38]. Profiles in Figure 3a–c show that about 69% of the electron density is confined into the InAs QW. Moreover, as the thickness of the top In_0.81_Ga_0.19_As barrier decreases, a non-negligible fraction of the electron densities extends up to the top surface (see red oval in Figure 3c). The 3D electron density at the surface is plotted as a function of *d* in Figure 3d (blue squares). Simulations show that this density is effectively zero for *d* ≥ 40 nm, while at *d* = 30 nm it rises to about 3.05 × 10^12^ cm^−3^ and then increases roughly exponentially with decreasing *d*, becoming more than 2 × 10^17^ cm^−3^ for InAs QWs placed directly on the surface. This increase correlates directly with the mobility decrease observed for *d* < 40 nm (Figure 1b) and confirms the role of surface scattering as the mechanism limiting electron mobility for shallow InAs 2DEGs [39]. Based on the above considerations, in the rest of this work we have selected a QW depth of 10 nm as the best compromise between preserving a high enough mobility and the requirements of interface transparency to a superconductive layer on the surface [19,29].

**Figure 4:**
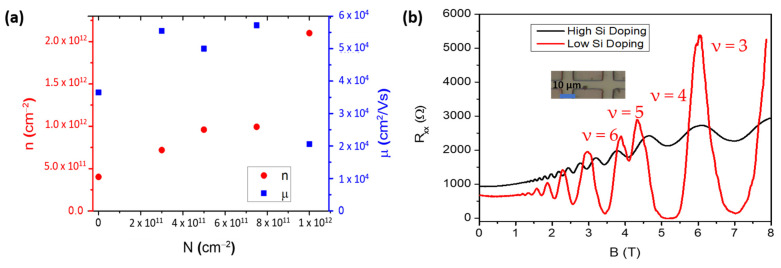
(**a**) Mobility and carrier density with respect to Si δ-doping density N, measured at 4 K on vdP structures. (**b**) Magnetotransport on non-gated Hall bars: longitudinal resistance (R_XX_) as a function of the out-of-plane magnetic field B at T=1.5 K for the samples with N = 1 × 10^12^ cm^−2^ (black) and N = 3 × 10^11^ cm^−2^ (red). Inset shows the optical photo of a similar Hall bar.

Remote Si doping is necessary to increase the carrier density in a controlled way in case higher densities than those forming naturally in this material system are needed. The tuning of electron density in shallow (10 nm) InAs 2DEGs was achieved through a Si δ-doping layer in the InAlAs barrier at a setback of 7 nm from the InGaAs/InAs QW. By changing the Si donor concentration N, we were able to tune *n* from 4 × 10^11^ cm^−2^ to more than 10^12^ cm^−2^ for *d* = 10 nm as shown in Figure 4a. Corresponding mobility µ increases up to ~ 6× 10^4^ cm^2^ /Vs for a doping concentration N = 7.5 × 10^11^ cm^−2^ and then it drops sharply for higher N.

**Figure 5:**
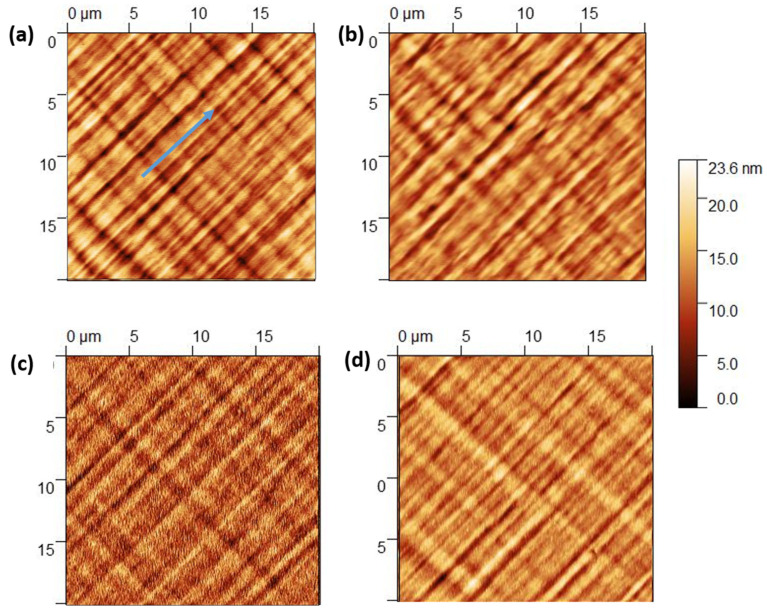
AFM scan of (**a**) 50 nm thick Al grown in situ on InAs/GaAs heterostructure sample (blue arrow indicates the [−110] direction), and samples (**b**) S-1, (**c**) S-2, and (**d**) S-3. The RMS roughness is 3.0 nm, 3.0 nm, 3.6 nm, and 3.3 nm, respectively.

Further analysis was completed through Shubnikov-de Haas (SdH) oscillations in a Hall bar geometry (Figure 4b). On the sample with N = 3 × 10^11^ cm^−2^ the SdH behaves as expected for a single-subband single-conducting channel, showing well-developed quantum Hall minima with Zeeman-split features at Landau level filling factors ν = 3, 5 (red curve) with corresponding plateaus in R_xy_ (not shown, see Ref. [40]). Note that the onset of oscillations happens at about 1T, which is only slightly higher than what we observed on deep InAs 2DEGs with much higher mobility (about 0.8T with 7.1 × 10^5^ cm^2^/Vs) [23]. Based on their period with 1/B, the charge density is close to the one measured with the classical Hall effect at low field (about 5.5 × 10^11^ cm^−2^). The mobility measured in Hall bar geometry here was about 8.6 × 10^4^ cm^2^/Vs, thus much higher than the corresponding one from vdP (and as high as 1.3 × 10^5^ cm^2^/Vs in another device). The discrepancy is possibly due to the inherent anisotropic nature of the scattering mechanisms present in metamorphic growth and to the associated cross-hatched morphology (see Figure 5 and Ref. [41]), which should become more evident in shallow 2DEGs, which are more affected by surface roughness effects. Since the Hall bar is oriented in the lower roughness [1$\bar{1}$0] direction, while the vdP geometry averages between the two <110>, the former may result in a higher electron mobility. On the sample with the highest silicon δ doping (N = 1 × 10^12^ cm^−2^), the oscillations do not go to zero and are superimposed on a strong background, while the carrier density from their period is 50% less than what we measure with the classical Hall effect. This suggests the formation of a parallel conductive channel at the Si δ-doping layer and explains the degradation of mobility and the surge of charge density for N = 1 × 10^12^ cm^−2^ in Figure 4a.

For a single density of N (5 × 10^11^ cm^−2^), we reduced the In_0.81_Ga_0.19_As top barrier thickness to 5 nm, which resulted in a decrease of electron mobility, as measured on vdP samples, from 5.5 × 10^4^ cm^2^/Vs to about 2.3 × 10^4^ cm^2^/Vs. This provides an option in case 2DEGs shallower than 10 nm are needed in order to increase interface transparency; hybrid devices containing 5 nm-deep InAs 2DEGs are currently under study.

### 3.2. Characterization of Al Thin Films and Devices

#### 3.2.1. Structural Characterizations

The morphological properties of the epitaxial surfaces were investigated by AFM. Aluminum grown in situ on an InAs QW (Figure 5a) exhibits an anisotropic cross-hatch pattern due to the underlying semiconductor and is typical for metamorphic growth [42]. The samples with Al grown directly on the GaAs layer also mimic the underlying semiconductor surface. The RMS surface roughness is 3.0 nm, very close to the values obtained on bare InGaAs [34], showing that the aluminum deposition forms a continuous 2D film conformal to the underlying semiconductor layers [43,44].

The samples with ex-situ Al layers: S-1, S-2, and S-3 (Figure 5b–d), are conformal to the underlying semiconductor as well and show similar cross-hatch patterns, with an RMS roughness comparable to or slightly higher than the in situ Al sample, although sputtered Al (S-2,3, Figure 5c,d) seems to exhibit a short-range granularity that is absent for MBE-deposited Al. Nevertheless, all our samples have a smoother surface morphology than those grown using different methods such as E-gun evaporation, where RMS roughness ~7–8 nm was observed [45].

**Figure 6:**
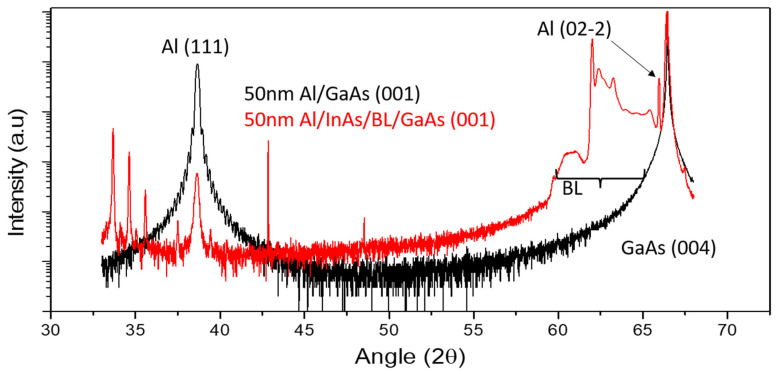
2θ XRD scans of 50-nm Al grown in situ on GaAs (black) and on InAs/GaAs heterostructures (red).

XRD analysis of the 50-nm Al film grown in situ on the InAs QW is compared to an equivalent film grown directly on epitaxial GaAs. Figure 6 shows symmetric (004) Bragg 2theta-omega scans for both samples obtained with synchrotron radiation at the MCX beamline, Elettra. In both cases, the main peak at 2ϴ = 66.45° comes from the GaAs substrate [46].

For Al grown on GaAs, only a single strong Al (111) peak at 38.14° is observed. Interference fringes confirm the film quality, along with the thickness of 50 nm. On the other hand, the sample grown on InAs, apart from the complex structure on the left of the GaAs (004) peak due to the InAlAs buffer and to the QW region marked as BL, shows two Al-related diffraction peaks: one at 38.14° corresponding to Al (111) and one at 65.2° (Al (02–2)). The intensity of Al (111) is about 100 times less than in the Al/GaAs (001) case and is likewise 2 orders of magnitude weaker than the (02-2) peak, despite its reference intensity being 3 times larger. This suggests that the Al film is mainly oriented in the (011) direction, with isolated (111) regions. Around the Al (111) peak, a few satellite peaks are visible, which may indicate the presence of defects. The lattice parameters inferred from the Al (111) peak are 7.028 Å on InAs and 7.025 Å on GaAs, consistent with the one of bulk Al (7.014 Å). Similarly, based on the (02-2) peak, the lattice parameter for the Al/InAs sample is 1.424 Å, very close to the bulk value of 1.431 Å, which indicates that the film is relaxed both on GaAs and in both domains on InAs.

**Figure 7:**
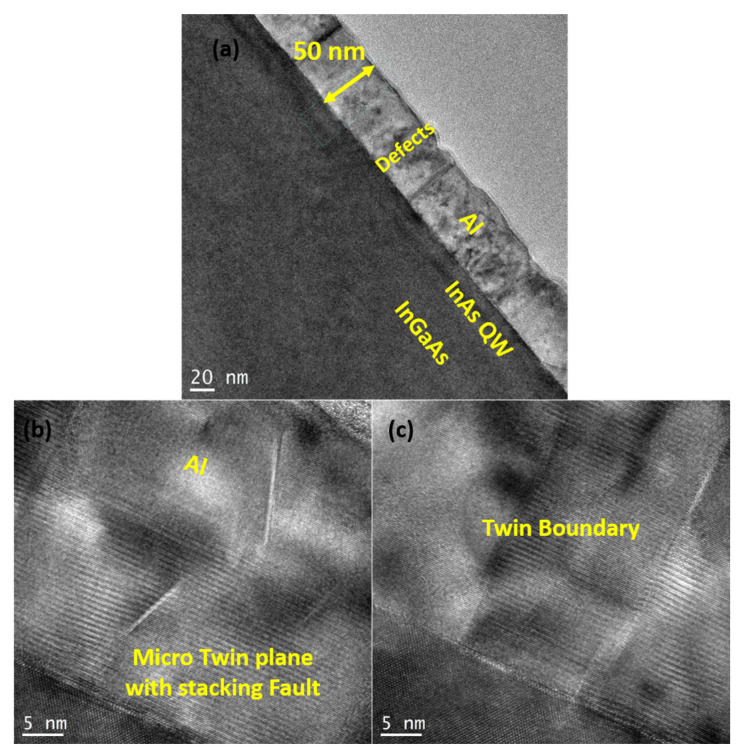
Bright-field TEM images of (**a**) 50-nm-thick Al grown on an InAs/InGaAs heterostructure, and (**b**) a micro twin plane with stacking faults and (**c**) twin boundaries in the Al layer grown on the InAs/InGaAs heterostructure.

More details about the structural and crystalline quality of the Al films were assessed by cross-sectional TEM measurements. The bright-field TEM image in Figure 7a depicts the growth of the in situ 50-nm Al layer on the InAs/InGaAs (001) QW, revealing the presence of defects within its structure. These defects are better identified in Figure 7b,c as twin planes, stacking faults, and twin boundaries.

**Figure 8:**
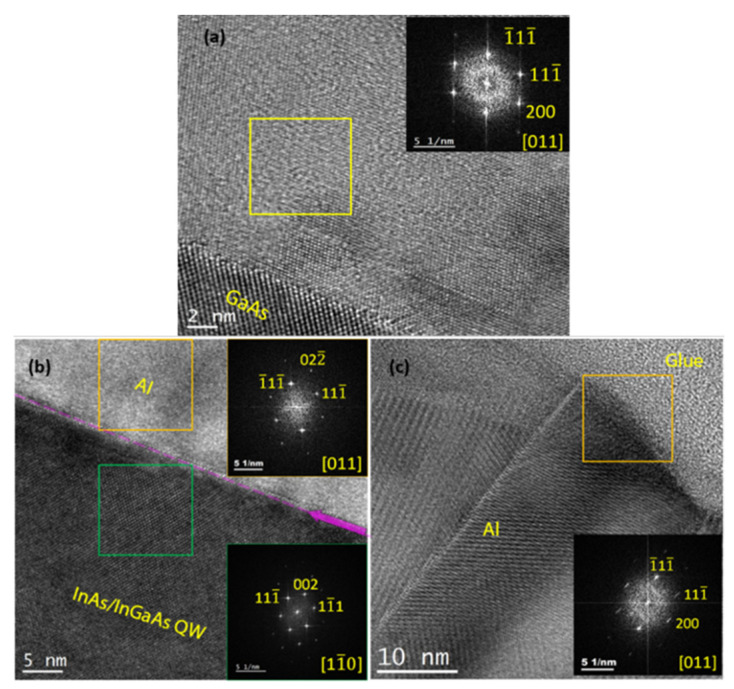
HRTEM images of a 50-nm Al layer grown on (**a**) GaAs (001) and (**b**,**c**) on InAs/InGaAs heterostructure, highlighting the high crystalline quality of the heterostructures. Insets within the figures show corresponding FFTs obtained from the regions marked with squares. The zone axis of Al layer is [110]. These FFTs reveal that the Al layer on the InAs/InGaAs heterostructure exhibits the out-of-plane directions along both (011) and (111).

A high-resolution TEM (HRTEM) image of the Al layer grown on GaAs is shown in Figure 8a, and images of Al on InAs/InGaAs (001) QW are displayed in Figure 8b,c, demonstrating the crystalline nature of the Al layer throughout its thickness. Furthermore, the interface between Al and the underlying semiconductor is found to be abrupt at the monolayer (ML) level. The smoothness of the semiconductor surface in the image indicates that the presence of the boundary is not associated with either a surface step or a crystallographic defect in the semiconductor.

To determine the orientation and strain of the Al layers, Fast-Fourier transform (FFT) patterns were obtained from the HRTEM images. The insets in Figure 8a–c display the corresponding FFTs obtained from the regions marked in the respective squares with the zone axis along the [110] direction. The FFT in Figure 8a illustrates that the Al layer on GaAs grows uniformly in the (111) orientation.

In contrast, the Al layer grown on the InAs/InGaAs QW exhibits an out-of-plane direction primarily oriented along (011) (Figure 8b). Upon closer inspection, we found the existence of isolated regions between twin boundaries growing along (111) (Figure 8c). Therefore, Al layers grown on InAs/InGaAs QW predominantly exhibit regions oriented along (011), with fewer regions aligned along (111). The lattice parameters, as calculated with FFT, are 7.02 Å for Al (111) both on GaAs and on InAs, and 1.45 Å for Al (02-2). These observations are consistent with the XRD analysis and confirm that all Al films are fully relaxed. In general, epitaxy of Al on GaAs is inherently constrained by the matching of the respective crystalline structures and lattice parameters, which set the elastic energy template. However, thermodynamic and kinetic limitations strongly affect the growth mode and orientation through controllable parameters such as Al deposition temperature and semiconductor surface stoichiometry and step density. This resulted in Al films oriented along (011), (001), (111), or in mixed-phase domains (see, e.g., [47]), depending on the growth conditions. In particular, films grown at about 0 °C on As-rich GaAs (001) resulted in (111) single crystals [45] or polycrystalline Al with (111) dominant orientation [47], while this orientation was not observed for films grown at higher temperatures or on As-stabilized GaAs surfaces [47] and references therein. This is consistent with our findings, since our Al (111) single crystals are obtained at about −50 °C on As-rich c(4 × 4) surfaces, obtained by cooling down the epitaxial GaAs films in As flux. Regarding Al/InAs films, our TEM and XRD results are consistent with the findings of Ref. [48], which revealed that the orientation of the Al layer switches from (111) to (011) when the in-plane lattice parameter of the underlying semiconductor exceeds 5.98 Å. Since in our case the in-plane lattice parameter is exactly the critical value, as inferred from the FFT analysis (see the pattern from the green area in Figure 8b), a mixture of the two orientations is observed. It is therefore reasonable to think that the lattice constant of the sample with the InAs QW is at or near the boundary between where (111) and (011) normal orientations in Al layers are favored [49]. The presence of defects (such as twin boundaries, dislocations, and stacking faults) within the Al layer observed by TEM (see Figure 7b,c) confirms that the occurrence of satellite peaks around the Al (111) peak in XRD analysis is defect-related [40]. Twin boundaries in Figure 7c and Figure 8c are characterized by their distinct, mirror-like symmetry (where the crystal lattice on one side of the boundary is a mirror image of the other), which sets them apart from other types of boundaries. This symmetry often results in a sharp, well-defined interface that can be observed as a continuous, straight line or plane within the crystal structure. Unlike other grain boundaries that may appear irregular or disordered, twin boundaries maintain a high degree of order and periodicity, making them easily identifiable in HRTEM images. Regarding the observed stacking faults, when Shockley partials of two extended dislocations on neighboring planes glide close together, their intrinsic stacking faults merge into an extrinsic stacking fault. This process can continue, and if a third dislocation joins the pair, the extrinsic stacking fault can transform into a two-layer microtwin.

#### 3.2.2. Electrical Characterizations

Four-terminal resistivity measurements of Al layers were completed on 1 × 4 mm^2^ samples from 4.2 K to 300 K with a testing current of 5 µA. Values of resistivity for a series of different Al films at 4 K and 300 K are shown in Table 1, together with the respective residual-resistance ratios (RRR), defined as R_300K_/R_4K_. Resistivities of 2.38 × 10^−9^ Ωm and 6.95 × 10^−9^ Ωm were achieved at 4 K for 50 nm and 10 nm, respectively (actual values should be even higher, especially for 10 nm, if one considers the 2–3-nm oxide layer forming on the Al surface [44]. Both resistivities and RRR increase with the Al layer thickness as size effects are less evident [50,51] and are comparable with those of state-of-the-art epitaxial Al layers deposited on GaAs [45,50], even though they are not single crystal domains. Moreover, our high RRR values are a signature of the film quality, and the temperature dependencies for T > 70 K up to room temperature exhibit the same linear behavior of single-domain Al epitaxial films [45].

In Table 1 we also compare samples S-1, S-2, and S-3. The resistivities of samples grown by sputtering (S-2 and S-3) are 1–2 orders of magnitude higher than those of samples grown in situ, and RRRs are less than 5. On the other hand, the electrical quality of the capped-decapped sample S-1 is comparable to that of direct in situ growth without air exposure. We conclude that ex-situ metal deposition through As capping-decapping of the semiconductor surface is a viable method to obtain hybrid systems with quality comparable to that of in situ grown ones and could find potential applications in case the employment of alternative superconductors, not available in the MBE chamber, is needed.

**Figure 9:**
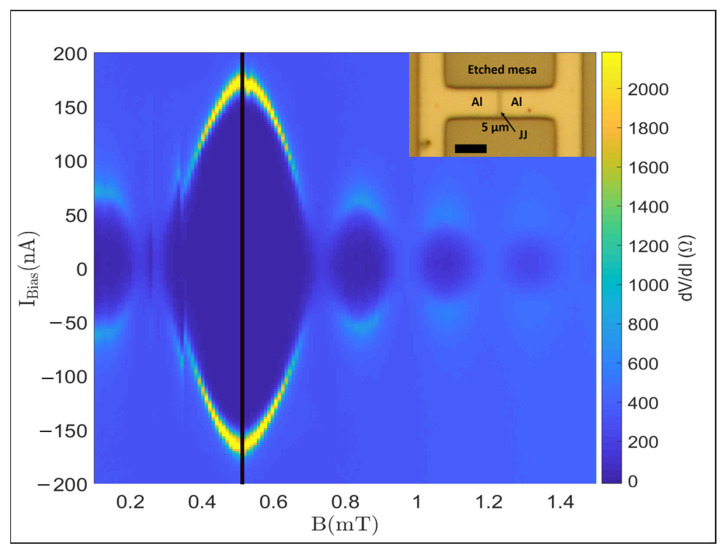
2D map of the differential resistance as a function of the magnetic field (x axis) and bias current (y axis) for a 500-nm-long Josephson Junction below 100 mK temperature.

As a benchmark of the quality of our hybrid systems, we obtained evidence of superconductivity due to the 2DEG being proximitized to the epitaxial Al layer in a Josephson junction. Figure 9 shows the map of the differential resistance as a function of bias current and magnetic field at <100 mK in a 500-nm-long junction, where a supercurrent and its modulation with magnetic field are clearly visible. The Fraunhofer-like interference pattern suggests homogeneous current flow in the junction. The middle of the central lobe is shifted from 0 T, which can be explained with a remanent magnetic field inside the magnet. The size and sign of this shift depend on the previous measurements and magnetic sweep directions. There is a cut taken at the middle of the central lobe, represented by the black line, corresponding to the effective 0 T. The critical current in this case is around 150 nA. We note that the period of the oscillations is shorter than expected. Based on the length L = 500 nm and width W = 5 μm, without taking into account the magnetic field penetration depth in Al, we expect the period of oscillations to be $\Delta B=\Phi_{0}/LW\approx 0.8\;mT$, where $\Phi_{0}=2.07\times 10^{-15}\;Wb$ is the flux quantum. However, the experimentally observed period is roughly half this value, likely due to the increased penetration depth in the thin Al film, effectively lengthening the junction [40,52,53].

## 4. Conclusions

In this work, we have shown that Al/InGaAs/InAs systems grown metamorphically on GaAs (001) substrates can provide state-of-the-art hybrid material platforms for topological superconductor devices in terms of crystal quality and electrical characteristics. We have studied the dependence of the low-temperature transport characteristics of the InAs 2DEG as a function of the QW depth and found that, despite a substantial increase of surface scattering effects, a proper choice of the top barrier thickness (around 10 nm) ensures maintaining a large enough mobility (up to 1.3 × 10^5^ cm^2^/Vs on delta-doped structures), while allowing at the same time the electron wave function to reach the surface. These transport properties compare favorably to those of state-of-the-art metamorphic shallow InAs 2DEGs grown on InP [25]. Thus, our shallow InAs QWs satisfy the requirements necessary to reach the topological superconducting regime by optimizing the mobility and distance of the QW from the surface and ensuring proximity effects.

Al layers were deposited in situ in the MBE system on clean, oxide-free InGaAs/InAs QWs. XRD and TEM analyses showed a double domain crystal structure, with dominant (011) phases and isolated (111) grains. Our films, however, possess electrical characteristics comparable to those of state-of-the-art single-crystal layers grown on GaAs and exhibit superconducting proximity effects in Josephson junctions. The observed phenomenology thus opens the way to the exploitation of hybrid qubits on GaAs-based technology.

## Figures and Tables

**Table 1:** Resistivity values at 4 K and 300 K for different samples grown during this work and compared with the state-of-the-art values [45,50].

Al Layer Thickness	ρ_4K_ (Ω·m)	ρ_300K_ (Ω·m)	RRR
10 nm on InAs/GaAs	6.95 × 10^−9^	2.22 × 10^−8^	3.19
50 nm on InAs/GaAs	2.38 × 10^−9^	3.05 × 10^−8^	12.8
50 nm on GaAs	3.14 × 10^−9^	2.21 × 10^−8^	7.03
S-1 (50 nm)	1.28 × 10^−9^	3.11 × 10^−8^	24.26
S-2 (50 nm, Sputtering)	3.76 × 10^−8^	1.24 × 10^−7^	3.29
S-3 (50 nm, Sputtering)	5.44 × 10^−8^	1.33 × 10^−7^	2.44
10 nm on GaAs (MBE) [50]	~4 × 10^−9^	~1 × 10^−8^	2.5
60 nm on GaAs (MBE) [45]	1.46 × 10^−9^	2.97 × 10^−8^	20.3

## Data Availability

The original data presented in the study are openly available in Zenodo at https://doi.org/10.5281/zenodo.14632201.
